# Histone Deacetylase 19 Controls Powdery Mildew Susceptibility by Attenuating Biosynthesis of Cuticular Wax and Salicylic Acid

**DOI:** 10.3390/jof12030178

**Published:** 2026-03-02

**Authors:** Mengdi Zhang, Wenrui Zhao, Pengfei Zhi, Haoyu Li, Cheng Chang

**Affiliations:** College of Life Sciences, Qingdao University, Qingdao 266071, China

**Keywords:** wheat, powdery mildew fungus, susceptibility, histone deacetylation, histone deacetylase 19, cuticular wax, salicylic acid

## Abstract

Phytopathogenic Ascomycetes *Blumeria graminis* f. sp. *tritici* (*Bgt*) causes wheat powdery mildew disease and impacts global wheat production. Decoding the molecular wheat-*Bgt* interaction could facilitate the wheat disease resistance breeding. In this study, we elucidated that wheat histone deacetylase 19 (TaHDA19) regulates susceptibility to *Bgt* pathogen by suppressing biosynthesis of cuticular wax and salicylic acid (SA). Knockdown of wheat *TaHDA19* gene expression led to in enhanced cuticular wax and SA accumulation, potentiated *Bgt* conidia germination and appressoria formation, attenuated formation of *Bgt* haustoria and microcolonies. Histone deacetylase TaHDA19 is enriched at the *TaECR* and *TaSARD1* promoter regions to facilitate histone deacetylation, and thus suppressing *TaECR* and *TaSARD1* transcription. In addition, we identified cuticular wax and SA regulated by TaHDA19 as chemical cues determining wheat pre- and postsusceptibility to *Bgt* pathogen. These findings collectively support that the wheat histone deacetylase TaHDA19 epigenetically suppresses cuticular wax and SA biosynthesis, thereby dampening chemical cues essential for the wheat powdery mildew susceptibility.

## 1. Introduction

Phytopathogenic Ascomycetes *Blumeria graminis* f. sp. *tritici* (*Bgt*) in the family Erysiphaceae infects bread wheat (*Triticum aestivum* L.), one of the most widely cultivated staple crop plants, to cause the devastating wheat powdery mildew disease [1]. The air-borne conidia of *Bgt* could infect wheat leaf blade, leaf sheath, stem, and spike to interfere with plant physiological processes and affect plant developmental events [2]. It was estimated that *Bgt* infection could cause above 5% loss of global wheat production [3]. The grain end-use quality of bread wheat is also dampened by the powdery mildew disease [1,2]. *Bgt* conidia germinates on the epidermal surface of wheat aerial organs like leaf blades and stems, undergoes the development of feeding structure, haustorium, in wheat epidermal cells, and culminates into the microcolony formation [1,2]. Decoding the molecular wheat-*Bgt* interaction is essential for the wheat breeding against powdery mildew disease.

As an important histone posttranslational modification (PTM), acetylation occurring at the histone N-terminal tail could neutralize the histone positive charge, weaken the histone-DNA association and facilitate gene transcription. Through removing the histone acetylation mark, histone deacetylases (HDAC) could suppress gene transcription [4,5,6]. Some histone deacetylases have been characterized in *Arabidopsis thaliana* to regulate plant development and response to environ-mental challenges. For instance, Arabidopsis histone deacetylase AtHDA9 represses the flowering activator gene *AGAMOUS-LIKE 19* (*AtAGL19*) in response to vernalization to regulate flowering time [7]. In addition, Arabidopsis histone deacetylase AtHDA9 function in concert with the SANT-domain containing protein POWERDRESS (PWR) and the transcription factor ABA-INSENSITIVE-4 (ABI4) to regulate drought stress response [8]. However, the function of histone deacetylases in cereal crops like the important staple crop bread wheat remains to be disclosed.

In this study, we explored the regulation of wheat histone deacetylase TaHDA19 on wheat powdery mildew susceptibility, and revealed that TaHDA19 mediates histone deacetylation at *TaECR* and *TaSARD1* promoters to suppresses cuticular wax and salicylic acid (SA) biosynthesis and thus finetune wheat susceptibility to *Bgt* pathogen. This study reported for the first time the important role of histone deacetylase TaHDA19 in the regulation of wheat powdery mildew susceptibility and provide important information for the wheat disease resistance breeding.

## 2. Materials and Methods

### 2.1. Wheat and Bgt Pathogen Maintenance

Seedlings of wheat cultivar Yannong 999 were cultivated in climate chambers under 16 h, 20 °C light/8 h, 18 °C dark cycle. *Bgt* isolate E09 was maintained on the seedlings of Yannong 999.

### 2.2. Gene Transcription Analysis

RT-qPCR and nuclear run-on assays were performed to analyze the transcript accumulation and transcription rate of genes as described [9,10,11]. *TaHDA19* gene is analyzed using primers 5′TTCAAGAGCGACCTCCTGA3′/5′AGATTCTCTCTTAACTCT3′.

### 2.3. Gene Silencing Assay

BSMV-VIGS and TIGS assays were performed to silence genes as described [9,10,11,12]. For the BSMV-VIGS, two independent silencing fragments targeting two conserved regions of *TaHDA19* gene were amplified using primers 5′AAGGAAGTTTAACGGAGCGGAGGAAGGAGA3′/5′AACCACCACCACCGTGTCTGCTACTTCTACGAC3′ and 5′AAGGAAGTTTAAAATAATCCCCAAACTTGTG3′/5′AACCACCACCACCGTGTCTTCGACGGCCTCTAC3′. For the TIGS, fragments of *TaHDA19* gene were amplified using primers 5′GGGGACAAGTTTGTACAAAAAAGCAGGCTTCCGGAGCGGAGGAAGGAGA3′/5′GGGGACCACTTTGTACAAGAAAGCTGGGTCGTCTGCTACTTCTACGAC3′.

### 2.4. Wheat-Bgt Interaction Analysis

*Bgt* conidial germination, appressorial formation, haustorial development and microcolony formation were statistically analyzed to judge the wheat-*Bgt* interaction as described [9,10,11]. *Bgt* conidial germination and appressorial formation rates were described as a percentage of *Bgt* conidia with germ tube development or appressorial formation. *Bgt* microcolony index were described as a percentage of germinated *Bgt* with microcolony formation. *Bgt* haustorium index was expressed as a percentage of *Bgt*-infected wheat cells with haustoria formation.

### 2.5. Cuticular Wax and Free SA Measurements

Measurement of cuticular wax and free SA in wheat leaves by gas chromatography-mass spectrometry (GC-MS) and high-performance liquid chromatography (HPLC) were conducted as described previously [9,10,11,13].

### 2.6. Wheat Leaf Cuticle Permeability Analysis

Water loss and chlorophyll leaching assays were conducted to judge the wheat leaf cuticle permeability as described [14,15].

### 2.7. Chromatin Immunoprecipitation (ChIP) Assay

For analyzing the TaHDA19 distribution at promoter regions of *TaECR* and *TaSARD1* genes, the ChIP assay was conducted using the wheat protoplast cells. We cotransfected the wheat protoplast with TaHDA19-HA and RNA interference (RNAi) constructs, and performed a ChIP assay to characterize the distribution of TaHDA19-HA at *TaECR* and *TaSARD1* promoters, and the coding sequence of *TaHDA19-6A* gene was employed as a representative *TaHDA19* gene for expressing the TaHDA19-HA fusion protein. For analyzing the histone acetylation H3K9ac and H3K14ac at *TaECR* and *TaSARD1* promoters, the ChIP assay was conducted using leaves of BSMV-VIGS wheat plants. We silenced all endogenous *TaHDA19* genes using BSMV-VIGS, and performed a ChIP assay to analyze H3K9ac and H3K14ac at *TaECR* and *TaSARD1* promoters. The ChIP assay was conducted as described [9,10,11,13].

### 2.8. Statistical Analysis

Three technical replicates for each assay were statistically analyzed by one-way ANOVA with Duncan’s post hoc test. bars with different letters are significantly different (*p* < 0.05), and letters were assigned in alphabetical order. Similar results were obtained from three independent biological replicates for these assays.

## 3. Results

### 3.1. Identification of Wheat TaHDA19 Genes

**Figure 1:**
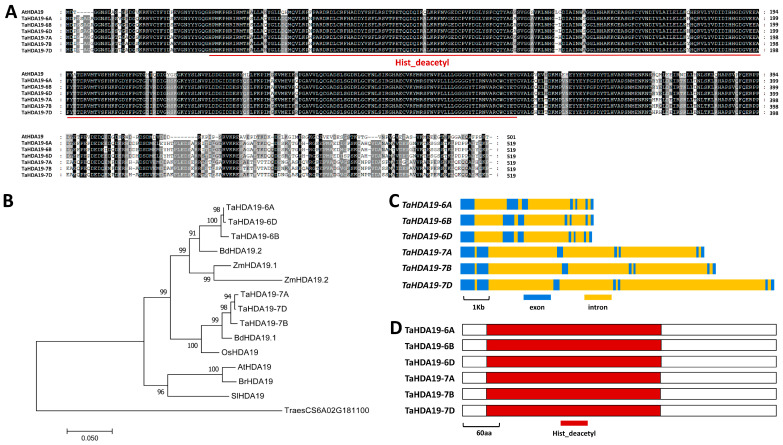
Identification and sequence analysis of wheat TaHDA19 proteins and genes. (**A**) Sequence alignments of Arabidopsis AtHDA19, wheat TaHDA19-6A, TaHDA19-6B, TaHDA19-6D, TaHDA19-7A, TaHDA19-7B, and TaHDA19-7D proteins. Same residues among 7 protein sequences are shaded in dark, while residues conserved in at least 4 of the 7 proteins are shaded in gray. (**B**) Phylogenetic analysis of the HDA19 proteins from plant species *Arabidopsis thaliana* (*At*), *Brassica rapa* (*Br*), *Solanum lycopersicum* (*Sl*), *Zea mays* (*Zm*), *Oryza sativa* (*Os*), *Brachypodium distachyon* (Bd), and *Triticum aestivum* (*Ta*). TraesCS6A02G181100 was employed as an external branch. (**C**) Exon and intron arrangement of wheat *TaHDA19-6A*, *TaHDA19-6B*, *TaHDA19-6D*, *TaHDA19-7A*, *TaHDA19-7B*, and *TaHDA19-7D* genes. (**D**) Domain characteristic of wheat TaHDA19-6A, TaHDA19-6B, TaHDA19-6D, TaHDA19-7A, TaHDA19-7B, and TaHDA19-7D proteins.

Through searching the wheat genome database using protein sequence of Arabidopsis histone deacetylase AtHDA19, we identified *TaHDA19* genes in wheat chromosomes 6A (*TraesCS6A02G184100*), 6B (*TraesCS6B02G212600*), 6D (*TraesCS6D02G171000*), 7A (*TraesCS7A02G365600*), 7B (*TraesCS7B02G261800*), and 7D (*TraesCS7D02G356800*) (Figure 1A). Phylogenetic tree reconstruction validated that these allelic TaHDA19 proteins are wheat close homologs of HDA19 proteins from *Arabidopsis thaliana* (*At*), *Brassica rapa* (*Br*), *Solanum lycopersicum* (*Sl*), *Zea mays* (*Zm*), *Oryza sativa* (*Os*), *Brachypodium distachyon* (*Bd*), and *Triticum aestivum* (*Ta*). TaHDA6 (*TraesCS6A02G181100*) protein sequence was employed as an external branch in the phylogenetic analysis (Figure 1B). As shown in Figure 1C, 7 exons and 6 introns exist in the genome sequence of allelic *TaHDA19* genes. Histone deacetylase (Hist_deacetyl) domain was identified from all TaHDA19 proteins (Figure 1D).

### 3.2. Regulation of TaHDA19 Gene on the Wheat Cuticular Wax and SA Biosynthesis and Powdery Mildew Susceptibility

**Figure 2:**
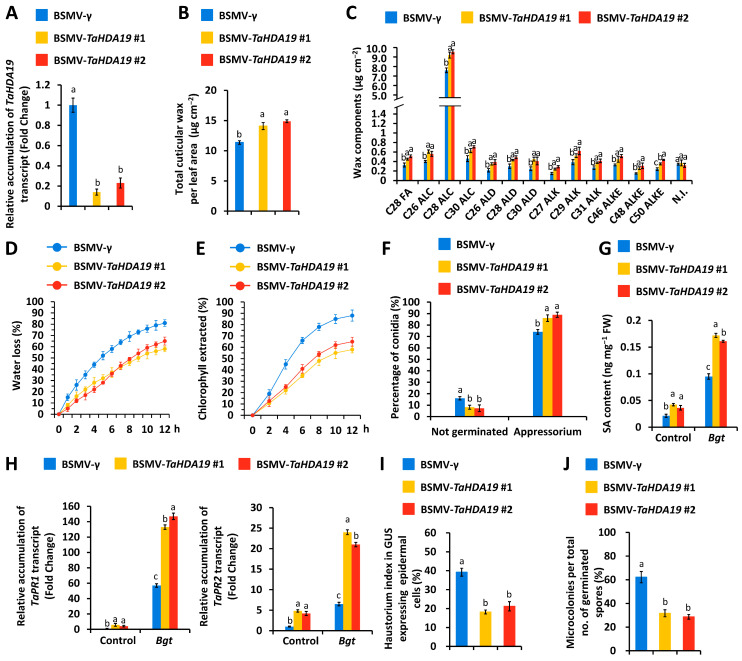
Functional characterization of wheat *TaHDA19* gene in the regulation of wax and SA biosynthesis, as well as compatible wheat–*Bgt* interaction. (**A**) Relative accumulation levels of *TaHDA19* gene transcript in the wheat leaves with silenced *TaHDA19* gene. (**B**) Total cuticular wax loads in the wheat leaves with silenced *TaHDA19* gene. (**C**) Accumulation of wax components VLC fatty acids (FA), alcohols (ALC), aldehydes (ALD), alkanes (ALK), alkyl esters (ALKE), and not identified compounds (N.I.) in the wheat leaves with silenced *TaHDA19* gene. (**D**) Water loss rates and (**E**) chlorophyll extraction levels analyzed in wheat leaves with silenced *TaHDA19* gene. (**F**) Statistical analysis of *Bgt* conidial germination and appressorium formation on wheat leaves with silenced *TaHDA19* gene. (**G**) Analysis of free SA accumulation in the wheat leaves with silenced *TaHDA19* gene. (**H**) Relative accumulation levels of *TaPR1* and *TaPR2* genes transcripts in the wheat leaves with silenced *TaHDA19* gene. (**I**) *Bgt* haustorial index analysis in wheat epidermal cells with silenced *TaHDA19* gene. (**J**) *Bgt* microcolony index analysis on wheat leaves with silenced *TaHDA19* gene. BSMV-γ was employed as the negative control, and data were statistically analyzed by one-way ANOVA with Duncan’s post hoc test (different letters indicate *p* < 0.05).

To analyze the function of *TaHDA19* genes in the compatible wheat-*Bgt* interaction, we conducted the BSMV-VIGS assay to silence all endogenous *TaHDA19* genes in the *Bgt*-susceptible wheat cultivar Yannong 999. As shown in the Figure 2A, accumulation levels of *TaHDA19* gene transcript were significantly reduced by BSMV-VIGS targeting two conserved regions of *TaHDA19* genes (BSMV-*TaHDA19* #1 and BSMV-*TaHDA19* #2). GC-MS results indicated that total cuticular wax loads increased from 11.29 μg cm^−2^ in the BSMV-γ control leaves to above 14.08 μg cm^−2^ in the *TaHDA19* gene-silenced wheat leaves (Figure 2B). As shown in the Figure 2C, accumulation of major wax constituents like VLC fatty acids (FA), alcohols (ALC), aldehydes (ALD), alkanes (ALK), alkyl esters (ALKE) were increased to different extent in the wheat leaves with silenced *TaHDA19* gene. Measurement of wheat leaf water loss rate and chlorophyll leaching levels revealed that cuticle permeability of wheat leaves decreased in the BSMV-*TaHDA19* #1 and BSMV-*TaHDA19* #2 plants, compared with BSMV-γ control plants (Figure 2D,E). We examined the *Bgt* prepenetration events, conidia germination and appressoria formation, on the *TaHDA19* gene-silenced wheat leaves. As shown in Figure 2F, approximately 8% more *Bgt* conidia germination and 12% more appressoria formation was observed on the wheat leaves with silenced *TaHDA19* gene. These results indicated that *TaHDA19* gene negatively regulate wheat cuticular wax biosynthesis and *Bgt* prepenetration events, conidia germination and appressoria formation.

We measured the levels of plant hormone salicylic acid (SA) in the *TaHDA19* gene-silenced wheat leaves. HPLC results showed that accumulation of free SA significantly increased in the wheat leaves with silenced *TaHDA19* gene (Figure 2G). As shown in the Figure 2H, accumulations of SA marker genes *TaPR1* and *TaPR2* transcripts were remarkable increased in the wheat leaves with silenced *TaHDA19* gene. We examined the *Bgt* postpenetration events, haustoria development and microcolony formation, on the *TaHDA19* gene-silenced wheat leaves. As shown in Figure 2I,J, approximately 17% less *Bgt* haustoria development and 23% less microcolony formation was observed on the wheat epidemral cells or leaves with silenced *TaHDA19* gene. These results indicated that *TaHDA19* gene negatively regulates wheat SA biosynthesis and facilitate *Bgt* postpenetration events, haustoria development and microcolony formation.

### 3.3. Distribution of Wheat Histone Deacetylase TaHDA19 Protein at Promoter Regions of TaECR and TaSARD1 Genes

**Figure 3:**
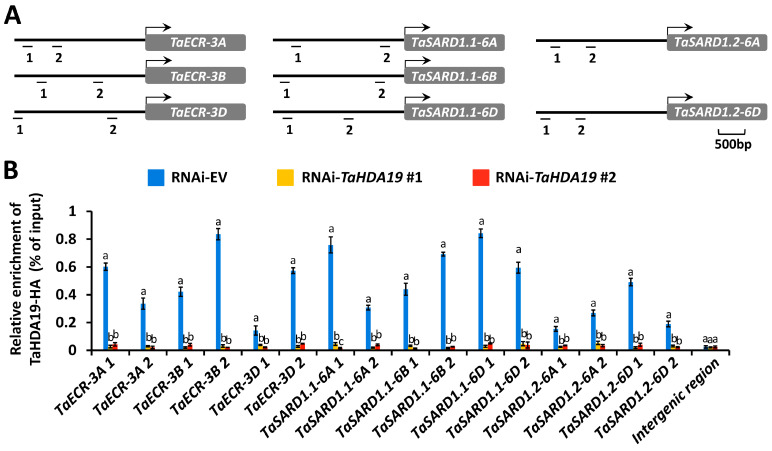
Analysis of the TaHDA19 distribution on the promoters of *TaECR* and *TaSARD1* genes. (**A**) Schematic diagram of *TaECR* and *TaSARD1* gene promoter regions subjected to the ChIP-qPCR analysis. (**B**) Distribution of the TaHDA19-HA protein on the promoters of *TaECR* and *TaSARD1* genes measured by the ChIP-qPCR assay. RNAi-EV was employed as the negative control, and data were statistically analyzed by one-way ANOVA with Duncan’s post hoc test (different letters indicate *p* < 0.05).

Previous studies revealed that wheat wax biosynthesis gene *TaECR* and SA biosynthesis activator gene *TaSARD1* are regulated by epigenetic events like histone acetylation [9,10,11,13]. We conducted the ChIP-qPCR assay to analyze the putative distribution of histone deacetylase TaHDA19 at promoter regions of *TaECR* and *TaSARD1* genes. We firstly expressed the fusion protein TaHDA19-HA in the wheat protoplast cell. Considering that wheat TaHDA19 isoforms shared high sequence identity, we employed allele *TaHDA19-6A* as a representative *TaHDA19* gene in the expression of the fusion protein TaHDA19-HA. *TaSARD1.2* gene was not identified from the wheat chromatin 6B in previous study [16], and *TaSARD1.2-6B* gene promoter was not included in this study. As shown in Figure 3, *TaECR* and *TaSARD1* promoter fragments were immuno-precipitated with the antibody against the fusion protein TaHDA19-HA, indicating that that wheat TaHDA19 protein occupied at promoter regions of *TaECR* and *TaSARD1* genes.

### 3.4. Epigenetic Regulation of Wheat Histone Deacetylase TaHDA19 on the Transcription of TaECR and TaSARD1 Genes

**Figure 4:**
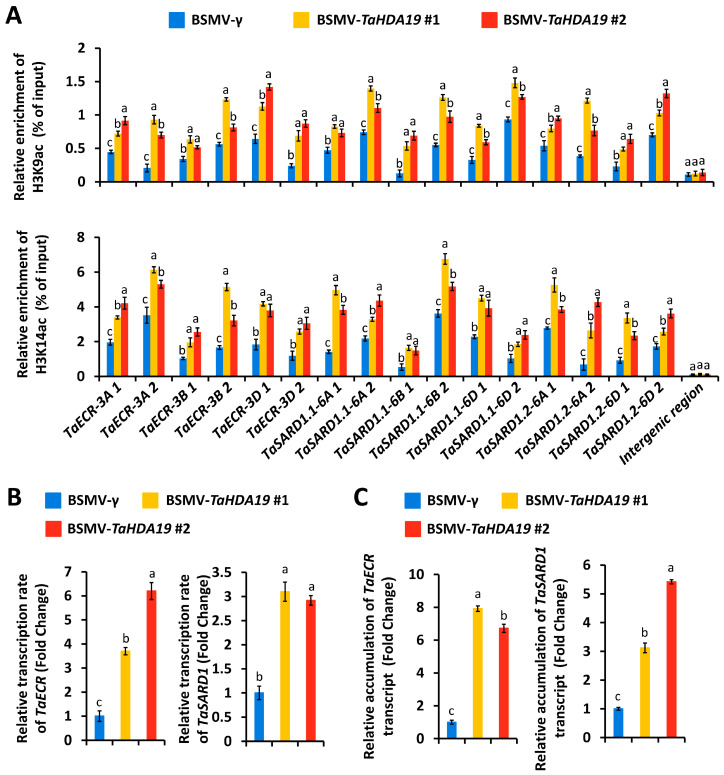
Analysis of histone acetylation and gene transcription at *TaECR* and *TaSARD1* loci in the wheat leaves with silenced *TaHDA19* gene. (**A**) Analysis of H3K9ac and H3K14ac levels at *TaECR* and *TaSARD1* gene promoters in the wheat leaves with silenced *TaHDA19* gene by ChIP-qPCR assay. Nuclear run-on (**B**) and RT-qPCR (**C**) analysis of *TaECR* and *TaSARD1* genes transcription rates and transcript accumulation in the wheat leaves with silenced *TaHDA19* gene. BSMV-γ was employed as the negative control, and data were statistically analyzed by one-way ANOVA with Duncan’s post hoc test (different letters indicate *p* < 0.05).

We conducted the ChIP-qPCR assay to analyze the putative regulation of histone deacetylase TaHDA19 on the histone acetylation at *TaECR* and *TaSARD1* promoters. The accumulation of histone acetylation marks H3K9ac and H3K14ac at *TaECR* and *TaSARD1* promoters were significantly increased in the *TaHDA19* gene-silenced wheat leaves (Figure 4A). These ChIP-qPCR results implicate that histone deacetylase TaHDA19 protein is enriched at *TaECR* and *TaSARD1* promoters to mediate histone deacetylation. We then performed the nuclear run-on assay to examine *TaHDA19* regulation on the *TaECR* and *TaSARD1* gene transcription. As shown in Figure 4B, transcription rates of *TaECR* and *TaSARD1* genes were remarkably enhanced in the *TaHDA19* gene-silenced wheat leaves. As revealed by the RT-qPCR analysis, accumulation levels of *TaECR* and *TaSARD1* transcript were significantly increased in the *TaHDA19* gene-silencing wheat leaves (Figure 4C). These data collectively suggested that histone deacetylase TaHDA19 directly suppresses of *TaECR* and *TaSARD1* gene transcription by promoting histone deacetylation at *TaECR* and *TaSARD1* promoters.

### 3.5. Functional Characterization of TaHDA19 and TaECR Genes in the Regulation of Wheat Cuticular Wax Biosynthesis and Bgt Prepenetration Susceptibility

**Figure 5:**
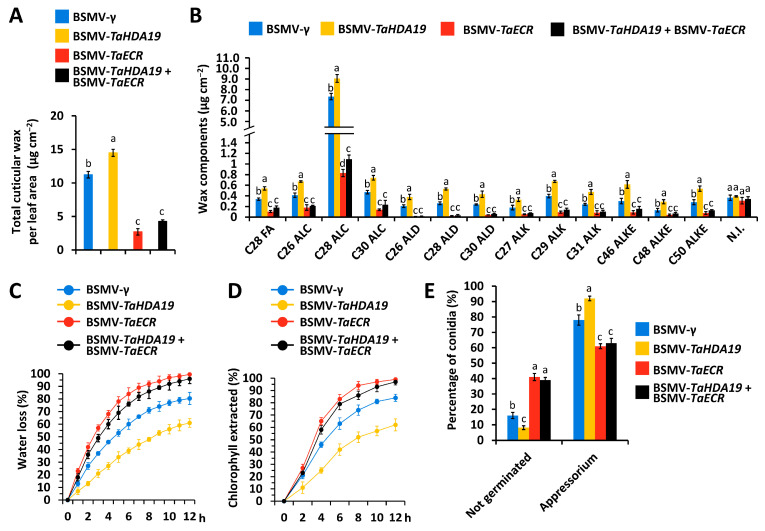
Functional characterization of *TaHDA19* and *TaECR* genes in the regulation of cuticular wax biosynthesis and *Bgt* prepenetration development. (**A**) Total cuticular wax loads in the wheat leaves with silenced *TaHDA19* or/and *TaECR* genes. (**B**) Accumulation of major wax components in the wheat leaves with silenced *TaHDA19* or/and *TaECR* genes. (**C**) Water loss rates and (**D**) chlorophyll extraction levels analyzed in the wheat leaves with silenced *TaHDA19* or/and *TaECR* genes. (**E**) Statistical analysis of *Bgt* conidial germination and appressorium formation on wheat leaves with silenced *TaHDA19* or/and *TaECR* genes. BSMV-γ was employed as the negative control, and data were statistically analyzed by one-way ANOVA with Duncan’s post hoc test (different letters indicate *p* < 0.05).

We simultaneously silenced *TaHDA19* and *TaECR* genes using BSMV-VIGS and analyzed the cuticular wax accumulation by GC-MS. As shown in Figure 5A, total cuticular wax loads significantly decreased from 11.23 μg cm^−2^ in the BSMV-γ control leaves and 14.41 μg cm^−2^ in the *TaHDA19* gene-silenced wheat leaves to 4.33 μg cm^−2^ in the wheat leaves with silenced *TaHDA19* and *TaECR* genes. Major wax constituents were remarkable decreased in the *TaHDA19*/*TaECR* genes co-silenced wheat leaves (Figure 5B). As shown in Figure 5C,D, cuticle permeability of wheat leaves obviously increased in the wheat plants with co-silenced *TaHDA19*/*TaECR* genes, compared with BSMV-γ control plants. We examined the *Bgt* prepenetration events, conidia germination and appressoria formation, on the *TaHDA19*/*TaECR* genes co-silenced wheat leaves. As shown in Figure 5E, significantly increased ration of *Bgt* conidia germination and appressoria formation was observed on the wheat leaves with co-silenced *TaHDA19*/*TaECR* genes, compared with control plants.

### 3.6. Functional Characterization of TaHDA19 and TaSARD1 Genes in the Regulation of Wheat SA Biosynthesis and Bgt Postpenetration Susceptibility

**Figure 6:**
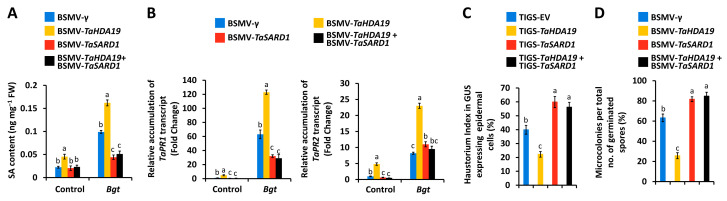
Functional characterization of *TaHDA19* and *TaSARD1* genes in the regulation of SA biosynthesis and *Bgt* postpenetration development. (**A**) Analysis of free SA accumulation in the wheat leaves with silenced *TaHDA19* or/and *TaSARD1* genes. (**B**) Relative accumulation levels of *TaPR1* and *TaPR2* genes transcripts in the wheat leaves with silenced *TaHDA19* or/and *TaSARD1* genes. (**C**) *Bgt* haustorial index analysis in wheat epidermal cells with silenced *TaHDA19* or/and *TaSARD1* genes. (**D**) *Bgt* microcolony index analysis on wheat leaves with silenced *TaHDA19* or/and *TaSARD1* genes. BSMV-γ and TIGS-EV were employed as the negative controls, and data were statistically analyzed by one-way ANOVA with Duncan’s post hoc test (different letters indicate *p* < 0.05).

We simultaneously silenced *TaHDA19* and *TaSARD1* genes using BSMV-VIGS and analyzed the SA accumulation by HPLC. As shown in Figure 5A, accumulation of free SA significantly decreased in the wheat leaves with co-silenced *TaHDA19* and *TaSARD1* genes, compared with control plants or *TaHDA19* gene-silenced plants (Figure 6A). Similarly, transcript levels of SA marker genes *TaPR1* and *TaPR2* were significantly decreased in the wheat leaves with silenced *TaHDA19* and *TaSARD1* genes, compared with BSMV-γ control leaves and *TaHDA19* gene-silenced. We examined the *Bgt* postpenetration events, haustoria development and microcolony formation, on the *TaHDA19*/*TaSARD1* genes co-silenced wheat epidermal cells or leaves. As shown in Figure 6C,D, significantly increased percentage of *Bgt* haustoria development and microcolony formation were observed on the wheat epidermal cells or leaves with silenced *TaHDA19* and *TaSARD1* genes, compared with controls and *TaHDA19* gene-silenced wheat epidermal cells or leaves.

## 4. Discussion

### 4.1. Wheat TaHDA19 Gene Suppresses Wax Biosynthesis to Dampen Bgt Prepenetraion Development

In this study, we identified allelic *TaHDA19* genes from wheat chromosomes 6A, 6B, 6D, 7A, 7B, and 7D as *AtHDA19* gene homologs in the agriculturally important crop bread wheat (*Triticum aestivum* L., AABBDD). Silencing of wheat *TaHDA19* gene resulted in enhanced cuticular wax accumulation, potentiated *Bgt* conidia germination and appressoria formation. Importantly, we demonstrated that histone deacetylase TaHDA19 protein is enriched at *TaECR* promoters to promote histone deacetylation and suppress *TaECR* gene transcription. Importantly, silencing of cuticular wax biosynthesis gene *TaECR* could dampen the cuticular wax accumulation and attenuate conidia germination and appressoria formation of *Bgt* pathogen on the *TaHDA19* gene-silenced wheat plants. These results implied that wheat histone deacetylase TaHDA19 protein epigenetically suppresses wax biosynthesis by promoting histone deacetylation at *TaECR* promoters, thereby negatively regulating biosynthesis of wheat cuticular wax essential for stimulating *Bgt* prepenetraion development events, conidia germination and appressoria formation.

Previous studies showed that the wheat Spt-Ada-Gcn5-acetyltransferase (SAGA) complex mediate the histone acetylation at *TaECR* promoters to activate the *TaECR* transcription and stimulate cuticular wax biosynthesis [13]. Herein, histone deacetylase TaHDA19 was demonstrated to promote histone deacetylation at *TaECR* gene promoters and attenuate cuticular wax biosynthesis. Therefore, it is intriguing the examine to the potential antagonistic action of SAGA complex and TaHDA19 in the regulation of *TaECR* gene transcription and cuticular wax biosynthesis. In the dicot model plant *A. thaliana*, AtHDA19 protein usually interacts with the transcriptional corepressor TOPLESS (TPL) to form the HDA19-TPL repressor complex in the regulation of plant flower development and hormone response [17,18]. Identifying the wheat TPL proteins might provide more insight into the mechanism underlying regulation cuticular wax biosynthesis. In addition, previous studies revealed that wax components VLC aldehydes could chemically stimulate conidia germination and appressoria formation of *B. graminis* [19,20]. In this study, VLC aldehydes overaccumulation and enhanced *Bgt* conidial germination were observed in the wheat leaves with silenced *TaHDA19* gene. Therefore, it is proposed that TaHDA19 negatively regulates *Bgt* conidia germination and appressoria formation probably via suppressing the production of wax cues VLC aldehydes.

### 4.2. Wheat TaHDA19 Gene Represses SA Biosynthesis to Facilitate Bgt Postpenetraion Development

Herein, we demonstrated that silencing of wheat *TaHDA19* gene resulted in enhanced SA accumulation, attenuated *Bgt* haustoria development and microcolony formation. Notably, we found that histone deacetylase TaHDA19 protein is enriched at *TaSARD1* promoters to promote histone deacetylation and epigenetically suppress *TaSARD1* gene transcription. Notably, silencing of SA biosynthesis activator gene *TaSARD1* could dampen the SA biosynthesis and potentiate *Bgt* haustoria development and microcolony formation in the *TaHDA19* gene-silenced wheat plants. These results suggested that wheat histone deacetylase TaHDA19 protein epigenetically suppresses SA biosynthesis by promoting histone deacetylation at the SA biosynthesis activator gene, thereby negatively regulating SA biosynthesis to facilitate *Bgt* postpenetraion development events, haustoria development and microcolony formation. Although we have measured the SA accumulation in the control (basal) and *Bgt*-induced (induced) wheat leaves in the *TaHDA19* gene-silenced BSMV-VIGS wheat leaves, constructing *TaHDA19* gene-knockout CRISPR mutant and analyzing SA accumulation in the time-course after *Bgt*-infection could provide more insight into the regulation of *TaHDA19* gene on wheat SA biosynthesis.

In *A. thaliana*, *AtHDA19* gene negatively regulate SA biosynthesis to maintain susceptibility to the hemibiotrophic pathogenic bacterium *Pseudomonas syringae* [21,22]. In this study, we demonstrated that *TaHDA19* gene suppresses SA biosynthesis to facilitate *Bgt* postpenetration susceptibility. These studies suggested that the suppression of *HDA19* gene on the SA biosynthesis might be conserved in the dicot Arabidopsis and monocot bread wheat. In addition, SWI/SNF chromatin remodeler TaSWI3B, TaSWI3D and DNA methyltransferase TaMET1 were identified as epigenetic suppressor of *TaSARD1* gene transcription and SA biosynthesis [9,10,11]. As summarized by prior reviews, different epigenetic regulators usually function in concert to regulate gene transcription. Characterizing potential interplays of histone deacetylase TaHDA19 protein with SWI/SNF type chromatin remodeler and DNA methyltransferase could provide novel insight into the epigenetic mechanism regulating SA biosynthesis.

**Figure 7:**
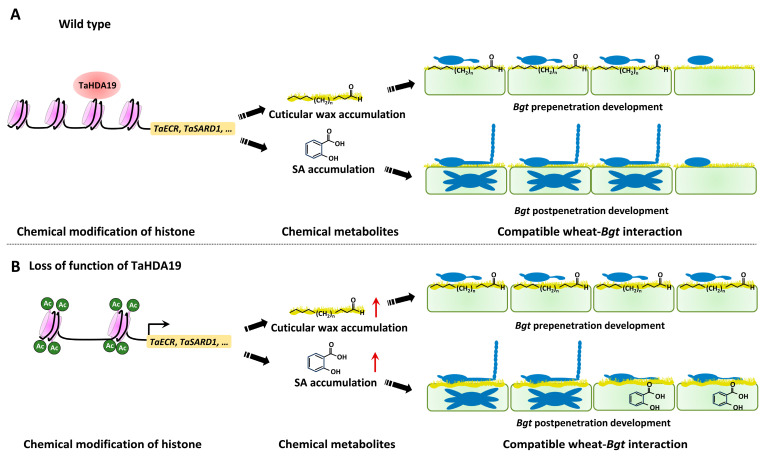
Proposed model for the regulation of wheat histone deacetylase TaHDA19 on SA and cuticular wax biosynthesis and powdery mildew susceptibility. (**A**) In the wild-type plants, wheat histone deacetylase TaHDA19 epigenetically suppresses biosynthesis of cuticular wax and SA by promoting histone deacetylation at the cuticular wax biosynthesis gene *TaECR* and SA biosynthesis activator gene *TaSARD1*. Cuticular wax stimulates *Bgt* prepenetraion development events, conidia germination and appressoria formation, whereas hormone SA initiates plant defense response and restricts *Bgt* postpenetraion development events, haustoria development and microcolony formation. (**B**) In the absence of wheat histone deacetylase TaHDA19, histone acetylation is potentiated at *TaECR* and *TaSARD1* promoters, which is associated with the epigenetic activation of *TaECR* and *TaSARD1* genes and enhanced cuticular wax and SA biosynthesis. As a result, *Bgt* pre- and postpenetration susceptibility was altered.

As depicted in Figure 7, we proposed a model of the wheat histone deacetylase TaHDA19 regulating the wheat powdery mildew susceptibility. In the wild-type plants (Figure 7A), wheat histone deacetylase TaHDA19 protein epigenetically suppresses biosynthesis of cuticular wax and SA by promoting histone deacetylation at promoters of *TaECR* and *TaSARD1* genes. Cuticular wax provides chemical cues stimulating *Bgt* prepenetraion development events, conidia germination and appressoria formation, whereas hormone SA initiates plant defense response and restricts *Bgt* postpenetraion development events, haustoria development and microcolony formation. In the absence of wheat histone deacetylase TaHDA19 (Figure 7B), histone acetylation is potentiated at *TaECR* and *TaSARD1* gene promoters, which is associated with the epigenetic activation of *TaECR* and *TaSARD1* genes and enhanced cuticular wax and SA biosynthesis. As a result, *Bgt* pre- and postpenetration susceptibility was altered.

Many questions are unaddressed in this study. For instance, causality between TaHDA19-mediated histone deacetylation and target gene transcriptional repression remains to be disclosed. Identifying TaHDA19 protein interacting partners by yeast-two hybrid and characterizing genome-wide targets of TaHDA19 protein by ChIP-seq could shed novel light into the gene transcriptional regulation by TaHDA19. Furthermore, although *TaHDA19*/*TaSARD1* and *TaHDA19*/*TaECR* genes co-silencing experiments support the pathway positioning of *TaHDA19* gene with *TaSARD1* and *TaECR* genes, pathway linearity and parallel regulation remains to revolved. Moreover, functional characterization of *TaHDA19* gene in wheat powdery mildew susceptibility was facilitated by BSMV-VIGS technique. Although two independent silencing fragments targeting different regions of *TaHDA19* gene were employed in the BSMV-VIGS assay, this technique has limitations like off-target silencing, transient nature, and effects across homoeologs. Generating stable transgenic *TaHDA19* gene-overexpression wheat plants or *TaHDA19* gene-knockout mutant might provide more insight into *TaHDA19* function and regulation in future research.

Multiple *Susceptibility* (*S*) genes that facilitate the wheat susceptibility to host-adapted pathogens have been identified [23,24,25]. Editing of these *S* genes by genome editing techniques like CRISPR-Cas9 and transcription activator-like effector nucleases (TALENs) could reverse susceptibility and confer disease resistance [26,27,28,29,30,31,32,33,34,35,36,37,38,39,40,41]. Although *TaHDA19* gene negatively regulates cuticular wax and SA biosynthesis to finetune pre-and post-penetration susceptibility to *Bgt* and could not be simply classified as a *S* gene, constructing the *TaHDA19*-knockout wheat plants by CRISPR-Cas9 and TILLING techniques might attenuate wheat post-penetration susceptibility to *Bgt* and could contribute to wheat breeding against powdery mildew in future research.

## 5. Conclusions

Herein, we characterized the function of wheat histone deacetylase gene *TaHDA19* in the regulation of compatible wheat-*Bgt* interaction. Our studies revealed that histone deacetylase TaHDA19 protein epigenetically suppressed biosynthesis of wheat cuticular wax and SA by promoting histone deacetylation at the promoter regions of wax biosynthesis gene *TaECR* and SA biosynthesis activator gene *TaSARD1*, thereby differentially affecting *Bgt* pre- and postpenetration events. These findings elucidated the novel epigenetic regulation of wheat chemical cues essential for the compatible wheat-*Bgt* interaction and provide valuable information for developing *Bgt*-resistant wheat varieties in future research.

## Data Availability

The original contributions presented in this study are included in the article. Further inquiries can be directed to the corresponding author.
